# Fast gaze reorientations by combined movements of the eye, head, trunk and lower extremities

**DOI:** 10.1007/s00221-015-4238-4

**Published:** 2015-03-12

**Authors:** Dimitri Anastasopoulos, J. Naushahi, Sokratis Sklavos, Adolfo M. Bronstein

**Affiliations:** 1 Academic Department of Neuro-Otology, Centre for Neuroscience, Imperial College London, Charing Cross Hospital, Fulham Palace Road, London, W6 8RF UK; 2 Department of Neurology, University of Ioannina, Ioannina, Greece

**Keywords:** Gaze shifts, Multisegmental coordination, Eye movements, Voluntary rotations

## Abstract

Large reorientations of the line of sight, involving combined rotations of the eyes, head, trunk and lower extremities, are executed either as fast single-step or as slow multiple-step gaze transfers. In order to obtain more insight into the mechanisms of gaze and multisegmental movement control, we have investigated time-optimal gaze shifts (i.e. with the instruction to move as fast as possible) during voluntary whole-body rotations to remembered targets up to 180° eccentricity performed by standing healthy humans in darkness. Fast, accurate, single-step movement patterns occurred in approximately 70 % of trials, i.e. considerably more frequently than in previous studies with the instruction to turn at freely chosen speed (30 %). Head-in-space velocity in these cases was significantly higher than during multiple-step transfers and displayed a conspicuously regular bell-shaped profile, increasing smoothly to a peak and then decreasing slowly until realignment with the target. Head-in-space acceleration was on average not different during reorientations to the different target eccentricities. In contrast, head-in-space velocity increased with target eccentricity due to the longer duration of the acceleration phase implemented during trials to more distant targets. Eye saccade amplitude approached the eye-in-orbit mechanical limit and was unrelated to eye/head velocity, duration or target eccentricity. Overall, the combined movement was stereotyped such that the first two principal components accounted for data variance almost up to gaze shift end, suggesting that the three mechanical degrees of freedom under consideration (eye-in-orbit, head-on-trunk and trunk-in-space) are on average reduced to two kinematic degrees of freedom (i.e. eye, head-in-space). Synchronous EMG activity in the anterior tibial and gastrocnemius muscles preceded the onset of eye rotation. Since the magnitude and timing of peak head-in-space velocity were scaled with target eccentricity and because head-on-trunk and trunk-in-space displacements were on average linearly correlated, we propose a separate controller for head-in-space movement, whereas the movement of the eye-in-space may be, in contrast, governed by global, i.e. gaze feedback. The rapid progression of the line of sight can be sustained, and the reactivation of the vestibulo-ocular reflex would be postponed, until gaze error approaches zero only in association with a strong head-in-space neural control signal.

## Introduction

Upright human subjects reorient to angularly distant targets (≥90°) by assembling fairly stereotypical, simultaneous, combined movements of the eye, head, trunk and lower extremities (Land 2004; Anastasopoulos et al. 2009). Such large reorientations to remembered targets can be accomplished as accurate, single-step shifts of the visual axis, covering at least 85 % of target eccentricity. Frequently, however, one or more short intervals of stationary gaze (i.e. of zero gaze velocity) emerge before target acquisition. In these cases, the initial segment of the gaze shift falls short of the target and more than 50 % of the target eccentricity is covered by a series of small saccades, presumably nystagmic fast phases, and head-in-space displacement (multiple-step gaze shifts; cat: Bergeron and Guitton 2002; humans: Anastasopoulos et al. 2009).

During the less time-efficient multiple-step gaze transfers, target acquisition takes at least 100–300 ms longer. It has been suggested that the higher the head-in-space velocity during this task, the more likely that a single-step gaze shift pattern will be elicited (Anastasopoulos et al. 2009). For example, patients with Parkinson’s disease and spasmodic torticollis generate accurate single-step gaze displacements to large target eccentricities less frequently than normal subjects, most likely because of the low head-in-space velocity which they can attain (Anastasopoulos et al. 2011, 2013), but this improves after deep brain stimulation treatment (Lohnes and Earhart 2012). However, the question of whether increasing head (and other body segments) velocity during whole-body gaze transfers will result in a higher proportion of single-step gaze shifts has never been directly investigated. In simple ecological terms is this single-step gaze transfer pattern the one we use when we want to look at an eccentric target fast?

Not only is the effect of an imposed time demand on the task not known; the exact kinematic or neural constraints which have to be fulfilled so that the more time-efficient single-step gaze shifts can be assembled are not known either. The initial segment (up to 90°) of such gaze reorientations is characterized by tight segmental coupling, as the three mechanical degrees of freedom available (i.e. eye-in-orbit, head-on-trunk and trunk-in-space) are reduced to two kinematic degrees of freedom (Sklavos et al. 2010). It is not, however, clear how this reduction is related to specific metric/kinematic measures of the coordinated movement.

A limitation of our previous studies is that data were obtained from relatively older subjects (Ss), turning at natural, freely chosen speeds (Anastasopoulos et al. 2009; Sklavos et al. 2010). Also, these studies assessed behaviour in a relatively small number of trials and only during the initial, brief segment of gaze progression. Thus, we do not know whether the reduction in the degrees of freedom prevails until target acquisition, given the increased contribution of the trunk in later stages of gaze shift (Sklavos et al. 2010). This is important because the trunk is the heaviest body segment and biomechanical challenges in controlling its motion accurately might interfere with the tight segmental coupling observed in the initial motion stages. In the current paper, we forced young subjects to execute the task as soon as possible and thus increase head-in-space velocity. In doing so, we expected to induce single-step gaze transfer patterns more frequently, and this should allow us to determine the statistical significance and degree of covariation among eye, head and trunk movements in late stages of gaze transfer, that is, almost until arriving on the target. Also, it can be expected that the dynamic trajectories of the involved body segments in such time-optimal task executions might reveal the time course and switching of the neural control signals of the underlying control mechanism; because of the instruction and the considerable head and trunk inertia, the magnitude of neural control signals to the effectors moving these segments is expected to be either maximal or minimal under these circumstances and switching between acceleration and deceleration sharply defined (‘bang–bang control’). Finally, the timing of segmental activation within this motor synergy was further evaluated by recording the initiation of electromyographic (EMG) activity from several muscles involved in stepping. A top-down sequence of segmental movement initiation has been observed when subjects turn on the spot in upright posture with the eye starting to rotate first and the foot last (Hollands et al. 2004), but data show that eye saccade initiation is considerably delayed when compared with data from sitting subjects (Scotto Di Cesare et al. 2013). Therefore, we hypothesized that this saccadic delay is introduced in order to optimize the coordination between the large number of rotating segments and the anticipatory postural adjustments required before the onset of movement. EMG recordings may disclose the timing and pattern of activation of several lower limb muscles in relation to eye rotation onset and spatial prediction.

## Materials and methods

### Subjects

We studied six healthy right-hand/leg dominant adults (three females), mean age 27 ± 1.2 years. No one wore spectacles, and selection ensured excellent physical condition. The study was approved by the Imperial College Riverside Ethics Committee in accordance with the Declaration of Helsinki, and all subjects provided informed consent.

### Protocol and apparatus

**Fig. 1:**
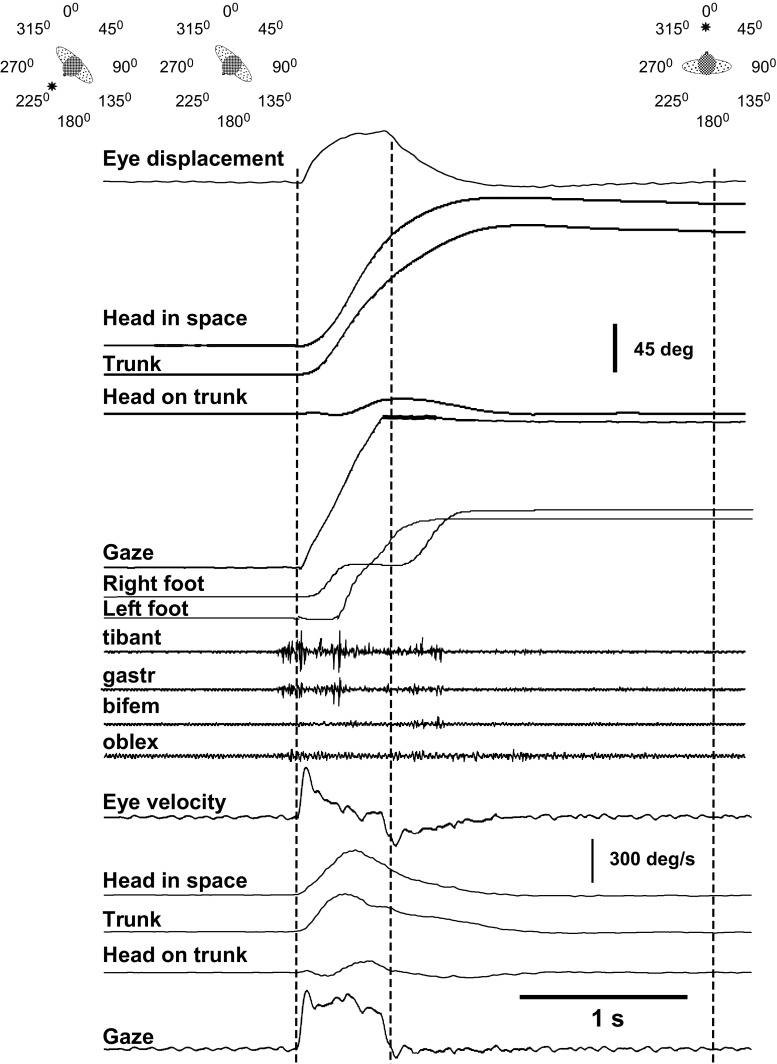
A rightward single-step gaze shift to the central remembered target at 135° offset in complete darkness. The *panels above* show a cartoon with the successive target presentations and head/trunk positions adopted before and after the combined movement. The *left* and *middle vertical dotted* markers indicate the onset and termination of the rapid displacement of the line of sight. Above and below the EMG recordings are shown position and velocity traces, respectively. *Note* the reacceleration in the eye velocity trace after the offset of the main eye saccade. Both during the acceleration and deceleration phases of head-in-space movement, changes in head-on-trunk velocity (second trace from the bottom) are complemented by variations of trunk-in-space velocity, such that a regular, approximately bell-shaped head-in-space velocity profile is maintained throughout the movement. Reappearance of the central LED is indicated by the *right vertical* marker. Synchronous EMG activity in the anterior tibial (tibant) and gastrocnemius (gastr) muscles precedes eye, head and trunk rotations

Details have been given previously (Anastasopoulos et al. 2009). In short, Ss stood in the centre of a circular array (radius 1.2 m) of eight LEDs, placed at 45° intervals at eye level, in darkness. At the beginning of each trial, they fixated and aligned their head, body and feet with the central LED. After a delay of 10 s, the central target was extinguished, thus indicating that another LED in one of the seven eccentric locations (45°, 90° and 135° either right or left of centre as well as at 180°) had been lit. The subject had to fixate, turn and align his body with the lit LED (outbound trials). After an interval of 15 s, the eccentric LED was turned off (Fig. 1, upper schematic), thus cueing subjects to return to the initial, central position (inbound or ‘return’ direction) and look in the direction of previously illuminated central LED. The central LED was, however, off during returning, and Ss moved in complete darkness (see below). Ss performed eighteen trials (outbound followed by return) to each LED location in random order.

It should be noted that trials to 45° targets are visually driven. For outbound trials to 90° or larger targets, the LED is initially not visible and Ss had no hint as to its location or in which direction they should turn. In inbound or ‘return’ trials, however, Ss knew where the centrally positioned target was and in this way the combined movement to the centre was exclusively memory driven (the central LED was turned on after additional 2.8 s).

In contrast to all our previous studies with this paradigm, where Ss were given no instruction as to the speed of the movement required, here Ss were instructed to accomplish turns as soon and as fast as possible.

Head-in-space, upper trunk and feet horizontal (yaw plane) movements were recorded using a Polhemus Fastrak motion analysis system (30 Hz; delay 4 ms, accuracy 0.15° RMS). Markers were placed on a light, adjustable helmet, on the spinous process of the seventh cervical vertebra and dorsally on each foot. Horizontal eye-in-orbit rotations were recorded using bi-temporal DC electrooculography (EOG) with a flat response to 90 Hz. EMG activity was collected by adhesive surface disc electrodes (CareFusion, Middleton, WI, USA, diameter 1 cm) placed at a distance of 3 cm near the motor point of the following muscles on the right side: anterior tibial, gastrocnemius, biceps femoris and external oblique.

### Data acquisition and analysis

The on–off LED signals, EOG, body position markers and EMG were sampled at 500 Hz and stored for offline analysis. A smoothing Savitzky–Golay filter was used for offline velocity measurements of the EOG position trace (MATLAB™ function *sgolayfilt*), while head and trunk velocities were calculated after subjecting the position signals to moving mean linear filtering (16 + 1, not weighted). Movement onset/offset for the various segments was calculated from the time derivative. Gaze (eye-in-space) displacement was obtained by adding EOG and head signals after appropriate scaling. Subtracting the trunk from the head signal resulted in an estimate of head-on-trunk movement. Biphasic deflections in the eye velocity trace were thought to represent blinks (Gandhi 2012); these trials were not further analysed. Raw EMG data were low-passed filtered at 10 Hz. Onset of EMG activity in relation to the onset of gaze shift (i.e. of the eye saccade) was defined visually (Fig. 1). To improve consistency, only rightward trials to initially non-visible targets (i.e. target eccentricity ≥90°, outbound and inbound) starting with the right foot were analysed for EMG onset.

#### ‘Within channels’ principal component (PC) analysis

We applied PC analysis to examine and quantify statistically the relationships among a set of 3-covarying variables (channels), i.e. eye-in-orbit, trunk-in-space and head-on-trunk, of *n*-observations by transforming the original data to a new set of 3-orthogonal variables, i.e. the PCs. The three PCs are derived in a decreasing order of importance (statistical significance), so that the first and the last PCs, respectively, account for maximal and minimal amount of variance in the original data. The PCs combine linearly with the original (elemental) variables, and if the three elemental variables are totally uncovaried, three statistically significant PCs will account for data set variability. In contrast, if two of the variables covary, then only two significant PCs account for data set variability and the space defined by the three elemental variables is reduced by one dimension, i.e. variation in the data set under consideration can be described by a plane (i.e. a two-dimensional coordinate system). Similarly, if all three variables covary, then only one significant PC accounts for data set variability and the space defined by the three variables is reduced by one further dimension (i.e. to a straight line). Thus, the number of significant PCs and the percentage of data variance they account for stand for the degree of covariation among the variables.

Percentages of the variation in the original set of variables, which the principal components account for, equal the normalized eigenvalues $\hat{\lambda }_{j} = \lambda_{j} /\sum\nolimits_{j = 1}^{3} {\lambda_{j} } ,\hat{\lambda }_{1} < \hat{\lambda }_{2} < \hat{\lambda }_{3}$ of the correlation matrix $\varvec{\varSigma}= {\text{Tr}}( \varvec{X}) \cdot \varvec{X}$ (Tr is for transpose) where

$$\varvec{X} = \left[ {\frac{{E^{(m)} - \mu \left( {E^{(m)} } \right)}}{{\sigma \left( {E^{(m)} } \right)}},\frac{{T^{(m)} - \mu \left( {T^{(m)} } \right)}}{{\sigma \left( {T^{(m)} } \right)}},\frac{{H_{\text{T}}^{(m)} - \mu \left( {H_{\text{T}}^{(m)} } \right)}}{{\sigma \left( {H_{\text{T}}^{(m)} } \right)}}} \right]_{n \times 3}$$

is the matrix of the data set with n rows (samples) and 3 columns (variables). $E^{(m)}$ represents eye, $T^{(m)}$ trunk and $H_{\text{T}}^{(m)}$ head-on-trunk angular displacement recorded as the *mth* sample of the trial, *m* = 1, …, *n*. For the purpose of PC analysis, raw position data were used (i.e. before filtering), and EOG was resampled at 30 Hz. Increasing time intervals, starting always with the onset of gaze, was analysed (‘sliding’ approach). Data centring and auto-scaling were carried out by subtracting the arithmetic means $\mu ( {E^{(m)} }),\mu ( {T^{(m)} }),\mu ( {H_{\text{T}}^{(m)} } )$ and dividing by the standard deviations $\sigma ( {E^{(m)} } ),\sigma ( {T^{(m)} }),\sigma ( {H_{\text{T}}^{(m)} })$, respectively. By multiplying the array of the data set consisting of the matrix $\varvec{X}_{n \times 3}$ with the array of eigenvectors (loadings, $\varvec{v}_{3 \times 3}$) of $\varvec{\varSigma}$, we get$\varvec{P} = \varvec{X} \cdot \varvec{v}$, where $\varvec{P}_{n \times 3}$ is the array of the scores of the PCs. These are organized in columns of an increasing order of importance, such that the third, second and first columns contain the first, second and third principal components, respectively, accounting for $\hat{\lambda }_{3} > \hat{\lambda }_{2} > \hat{\lambda }_{1}$ of the variance of the original variables, respectively. Then, the contribution of the $\varvec{X}^{{( \varvec{i})}}$ original variable to the $\varvec{P}^{( j )}$ principal component (weight) is $v_{ij}$. The loadings of the three variables define the new coordinate system.

Eigenvalues $( {\lambda_{j} }),\,j = 1,2,3$ and their corresponding eigenvectors $( {\varvec{v}_{j} }),\,j = 1,2,3$ were computed by using the MATLAB™ function *eig*. For each data set (trial), the eigenvalue $\lambda_{j}^{(k)}$ and weights $v_{ij}^{(k)}$, *k* = 1, …, *N*, are calculated. Further, by averaging across all trials, the mean eigenvalue $\bar{\lambda }_{j} = \sum\nolimits_{k = 1}^{N} {\lambda_{j}^{(k)} } /N$ and mean weights $\bar{v}_{ij} = \sum\nolimits_{k = 1}^{N} {v_{ij}^{(k)} } /N$ have been calculated.

#### PCs’ statistics

*Bartlett’s test of sphericity* was used to validate the hypothesis that the first principal component was significantly different from all those of lower importance, i.e. both second and third. This has to be accepted if the test statistic function $(n - 1)\cdot\sum\nolimits_{i = 1}^{j - 1} {\log (\lambda_{i} )} + (n - 1)\cdot(j - 1)\cdot\log ((j - 1)^{ - 1} \sum\nolimits_{i = 1}^{j - 1} {\lambda_{i} } )$ is greater than the critical value of Chi-square distribution with $\frac{1}{2}(j - 2)(j + 1)$ degrees of freedom. As Bartlett’s test is insufficient for testing the significance of the last two principal components, i.e. those of the lowest importance (*j* = 1, 2), a test for proportion of total variance was applied post hoc. A desired proportion of total variance at 99.0 % was selected and excluded all principal components, which accounted for <1 % of the total variance; one-sample independent *t* test was performed for the arithmetic means of variance proportion of second and third PCs and tested whether they were significantly >1 %. When normality failed according to a one-sample Kolmogorov–Smirnov nonparametric test, a binomial nonparametric test for the median value was carried out instead.

#### Multilinear regression

Average eye-in-orbit ($\varvec{E}^{(a)} = \frac{1}{N}\sum\nolimits_{j = 1}^{N} {\varvec{E}^{( j)} }$), head-on-trunk ($\varvec{H}_{\text{T}}^{(a)} = \frac{1}{N}\sum\nolimits_{j = 1}^{N} {\varvec{H}_{\text{T}}^{(j)} }$) and trunk ($\varvec{T}^{(a)} = \frac{1}{N}\sum\nolimits_{j = 1}^{N} {\varvec{T}^{{^{( j )} }} }$) displacement trajectories across all *N*-trials, separately for various target eccentricities and time intervals from gaze onset (Table 1), were calculated. Any point of trunk displacement trajectory $T_{i}^{(a)}$ ($i = 1, \ldots ,n$) is represented as a linear combination of $E_{i}^{(a)}$, $H_{{{\text{T}},i}}^{(a)}$: $T_{i}^{(a)} = b_{0} + b_{1} \cdot E_{i}^{(a)} + b_{2} \cdot H_{i}^{(a)} + \varepsilon_{i}$ where $\varepsilon_{i}$ are fitting standard errors. This can be written as a matrix equation by introducing vectors $\varvec{b}_{3 \times 1} = \text{Tr}( {b_{0} ,b_{1} ,b_{2} } )$, $\varvec{\varepsilon}_{n \times 1} = ( {\varepsilon_{i} } )$, $\varvec{1}_{n \times 1} = \text{Tr}( {1, \ldots ,1})$ and the matrix $\varvec{A}_{n \times 3} = [ {\varvec{1},{\varvec{E}}^{(a)}, \varvec{H}_{\varvec{T}}^{(a)} }]$: $\varvec{T}^{(a)} = \varvec{A} \cdot \varvec{b} +\varvec{\varepsilon}$. The estimate of $\varvec{b}$ which minimizes the sum square error ${\rm T}\text{r}(\varvec{\varepsilon}) \cdot\varvec{\varepsilon}$, as in multiple linear regression, is then given by $\varvec{\hat{b}}=({\rm T}\text{r}(\varvec{A}) \cdot \varvec{A})^{ - 1} \cdot ({\rm T}\text{r}(\varvec{A}) \cdot \varvec{T}^{(a)} )$. The new estimate of trunk displacement trajectory is $\hat{\varvec{T}}^{(a)} = \varvec{A} \cdot \hat{\varvec{b}}$ and, in principle, the 3D vector $(\varvec{E}^{(a)}, \varvec{H}_{\text{T}}^{(a)} ,\hat{\varvec{T}}^{(a)} )$ lies on a surface plane. The coefficient of determination $R^{2} = {\text{Tr}}(\hat{\varvec{T}}^{(a)} - \varvec{T}^{(a)} ) \cdot (\hat{\varvec{T}}^{(a)} - \varvec{T}^{(a)} )/(n - 3)$ is an estimate of the goodness of fit of the surface plane to the vector of displacement trajectories. Finally, $\theta = \tan^{ - 1} (\hat{b}_{1} ) + \frac{\pi }{2},\phi = \tan^{ - 1} (\hat{b}_{2} )$ are the angles of orientation (in radians) of the surface plane with respect to trunk-in-space/head-on-trunk and head-on-trunk/eye-in-orbit planes, respectively.

**Table 1:** Covariation of eye-in-orbit, head-on trunk and trunk-in-space displacements

*T* (ms)	90°	135°	180°
0–165	PCs: 91.7 ± 6.5, 7.9 ± 6.4, 0.4 ± 0.5, *φ* = 52, *θ* = 90 *N* = 68 *G* = 52.2 ± 12.3	PCs: 91 ± 6.2, 8.5 ± 6.1 0.4 ± 0.9 *φ* = 42, *θ* = 92 *N* = 75, *G* = 57.1 ± 13.4	PCs: 95.1 ± 3.6, 4.7 ± 3.6 0.2 ± 0.2 *φ* = 46, *θ* = 96 *N* = 18, *G* = 49.4 ± 6.2
0–264	PCs: 89 ± 5.4, 10.3 ± 5.2 0.6 ± 0.9 *φ* = 57, *θ* = 89 *N* = 61, *G* = 72 ± 12.7	PCs: 87.7 ± 5.3, 11.8 ± 5.1 0.5 ± 0.7 *φ* = 48, *θ* = 91 *N* = 73, *G* = 86.1 ± 20.2	PCs: 89.1 ± 11.4, 10 ± 11.2 0.4 ± 0.4 *φ* = 52, *θ* = 96 *N* = 18, *G* = 71.5 ± 18.1
0–333	PCs: 89.3 ± 5.6, 10.2 ± 5.6 0.5 ± 0.6 *φ* = 66, *θ* = 92 *N* = 41, *G* = 82.3 ± 13.6	PCs: 87.2 ± 6.5, 12.2 ± 6.2 0.7 ± 1.0 *φ* = 60, *θ* = 87 *N* = 53, *G* = 107.1 ± 23.8	PCs: 88.7 ± 9.0, 10.9 ± 9.6 0.4 ± 0.4 *φ* = 58, *θ* = 91 *N* = 18, *G* = 96.7 ± 18.8
0–396	PCs: 86.4 ± 7, 13 ± 7.2 0.6 ± 0.8 *φ* = 72, *θ* = 90 *N* = 15, *G* = 84.0 ± 5.7	PCs: 86.5 ± 7.3, 12.8 ± 6.9 0.7 ± 0.9 *φ* = 70, *θ* = 87 *N* = 43, *G* = 117.1 ± 20.8	PCs: 88.3 ± 9.1, 11.1 ± 8.7 0.5 ± 0.6 *φ* = 60, *θ* = 90 *N* = 18, *G* = 120.5 ± 23.2
0–495		PCs: 85.7 ± 6.8, 13.6 ± 6.4 0.7 ± 0.9 *φ* = 70, *θ* = 96 *N* = 27, *G* = 125.2 ± 19.8	PCs: 86.9 ± 10.6, 12.6 ± 9.1 0.5 ± 0.6 *φ* = 66, *θ* = 82 *N* = 16, *G* = 141.2 ± 25.6

Segmental coupling is quantified by the percentage (%) of variance accounted for by principal components. The first and second PCs were overall significant (*p* < 0.001). The third PC accounted for <1 % of displacement variance and was non-significant (*p* > 0.1). *T* time interval analysed during single-step gaze transfers separately to 90°, 135° and 180° targets, starting always from gaze onset; *N*, number of trials analysed; *G*, mean amplitude ± SD in degrees, of gaze displacement during the time interval under consideration. The orientation of the covariation plane is indicated by the angles *φ* (in degrees, with respect to the plane defined by the axes eye-in-orbit/head-on-trunk) and *θ* (with respect to the plane defined by the axes trunk-in-space/head-on-trunk). The coefficient of determination *R* ^2^ was always >96 %

## Results

### Patterns of voluntary reorientations

**Fig. 2:**
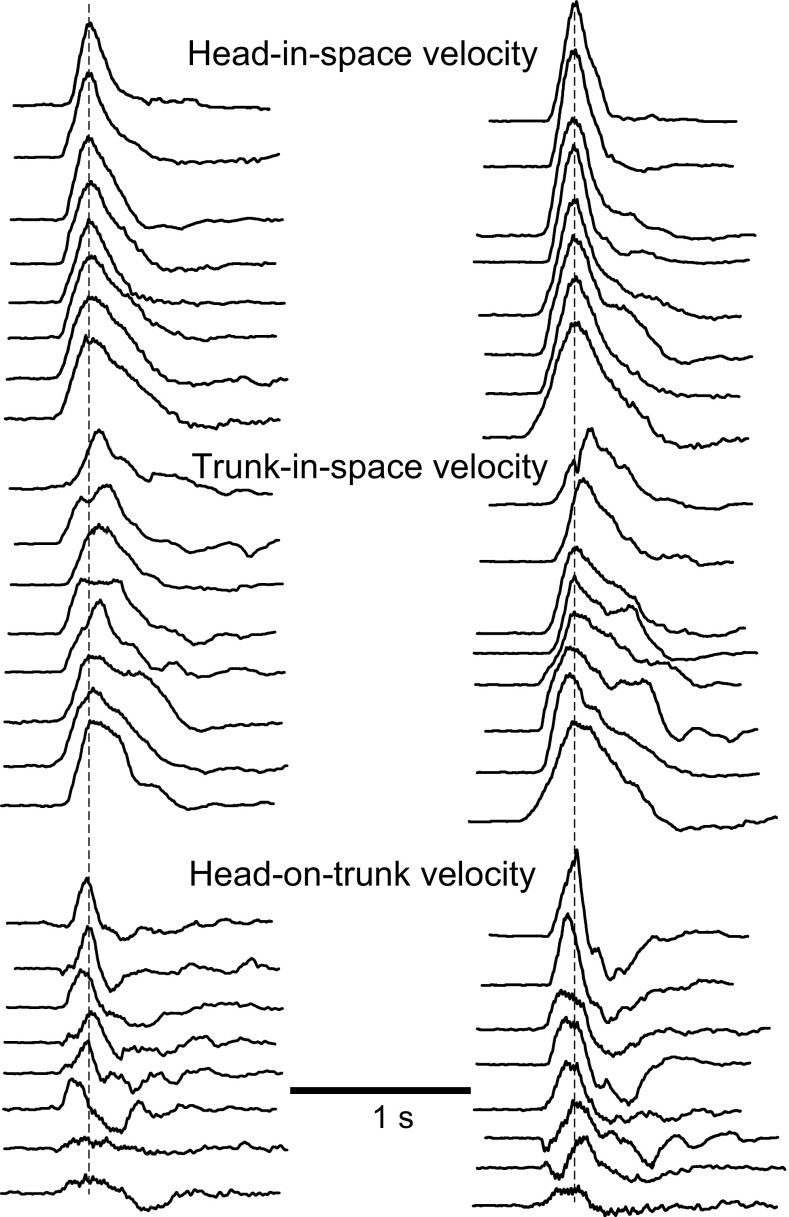
A family of eight superimposed head-in-space velocity trajectories from single-step gaze shifts. Displacements to 90° (*left*) and 135° (*right*) eccentricity targets (*upper rows*) and the corresponding trajectories of trunk-in-space (*middle rows*) and head-on-trunk velocity (*lower rows*) from the same trials. All of the traces are aligned on the peak of head-in-space velocity and normalized such that head-in-space velocity has in all cases the same arbitrary value. They are ranked according to the displacement magnitude of peak head-on-trunk deflection. *Note* how the cooperative or opposing motion of head-on-trunk and trunk-in-space produces in all cases a regular, approximately bell-shaped velocity profile of head-in-space movement

**Fig. 3:**
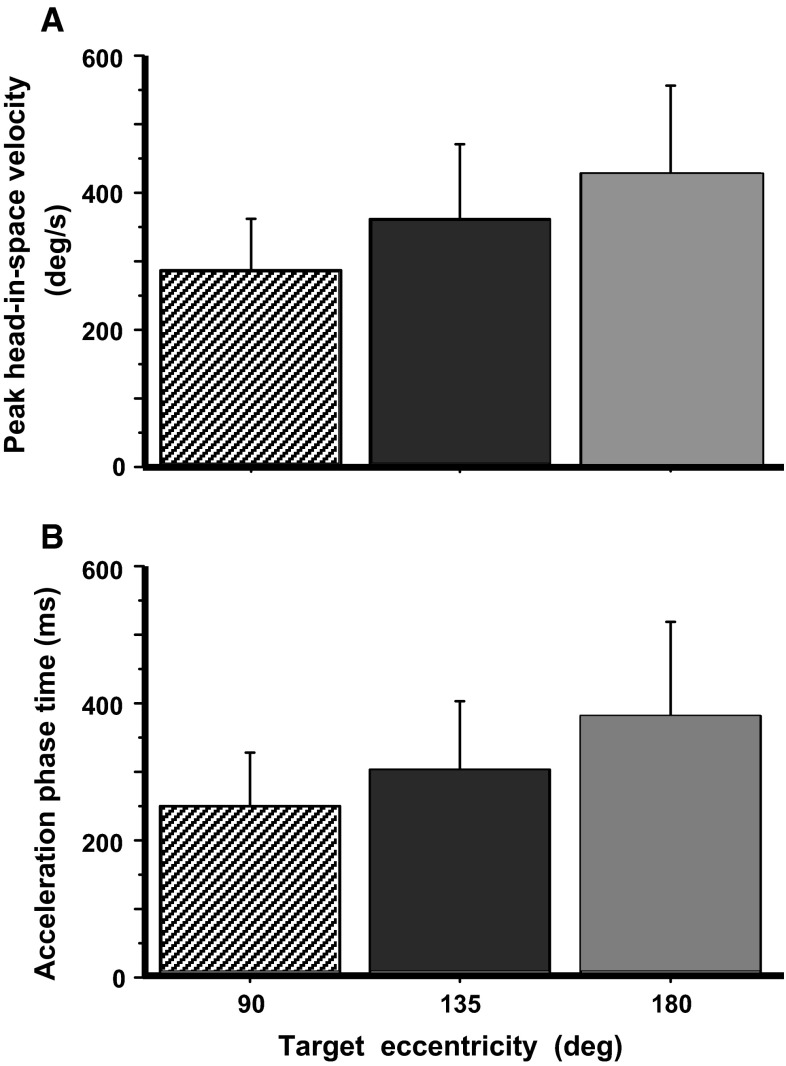
Kinematics of head-in-space movement. Though quite variable, peak head-in-space velocity and acceleration time interval increase significantly with target eccentricity

Details of the movement pattern in normal subjects moving at freely chosen speed have been given and illustrated extensively in earlier publications (Figs. 1, 2, 3, Anastasopoulos et al. 2009; Figs. 1, 2, 3, Sklavos et al. 2010; Fig. 2, Anastasopoulos et al. 2011; Fig. 1, Anastasopoulos et al. 2013).

In the outbound condition, particularly of ≥90° amplitude and, hence, of unpredictable target location, the prevailing movement pattern involved termination of the primary gaze shift well before target acquisition. Compensatory, slow-phase eye movements would rotate the eye centrally, and one or more intervals of stationary gaze, due to the vestibulo-ocular reflex, would appear (multiple-step gaze shifts as defined in our previous publications). Outbound turns and visually guided gaze shifts (i.e. to ±45° visual targets) will not be considered further here.

Inbound or ‘return’ reorientations to targets ≥90° consisted predominantly of single-step displacements of the visual axis covering at least 85 % of target eccentricity (Fig. 1). Here, we concentrate mainly on the description of these responses to the centrally located, remembered target and try to identify the kinematic constraints allowing for the tight segmental coupling and the execution of such quick and accurate gaze saccades. Single-step movement patterns occurred in 82, 78 and 56 % of inbound trials to 90°, 135° and 180° target eccentricities, respectively, i.e. considerably more frequently than in our previous studies with the instruction to turn at freely chosen speed (60, 35 and 25 %, respectively).

### Metrics and kinematics of time-optimal, single-step gaze transfers

As already reported in the literature for large saccades with the head free to move (reviewed by Freedman 2008), peak eye saccade velocity (448 ± 135°/s, mean ± SD) and duration (261 ± 87 ms) were unrelated to eye saccade amplitude (46.8 ± 5.6° on average, approaching thus the mechanical eye-in-orbit limit). An example executed under the current ‘as fast as possible’ instruction is shown in Fig. 1. In most instances, the eye saccade velocity profiles had a single peak. In several occasions, however, a second, considerably smaller, peak was discernible shortly before eye saccade offset. In other instances, one or two tiny eye saccades (of <4° amplitude) appeared before gaze shift end (Fig. 1). These reaccelerations occurred almost always after the velocity of head-in-space motion had begun to decrease but, still, before gaze end (see below).

Gaze transfer was terminated by the emergence of VOR (at mean amplitude 84.6 ± 5.0°, 131.6 ± 8.1° and 172.1 ± 9.8°; unsigned errors 6.4°, 7.1° and 9.4° for 90°, 135° and 180° target eccentricity, respectively), and the line of sight was stabilized, in darkness, quite near the memorized centrally located target. Ss sometimes made a corrective small saccade after the LED was turned on.

**Fig. 4:**
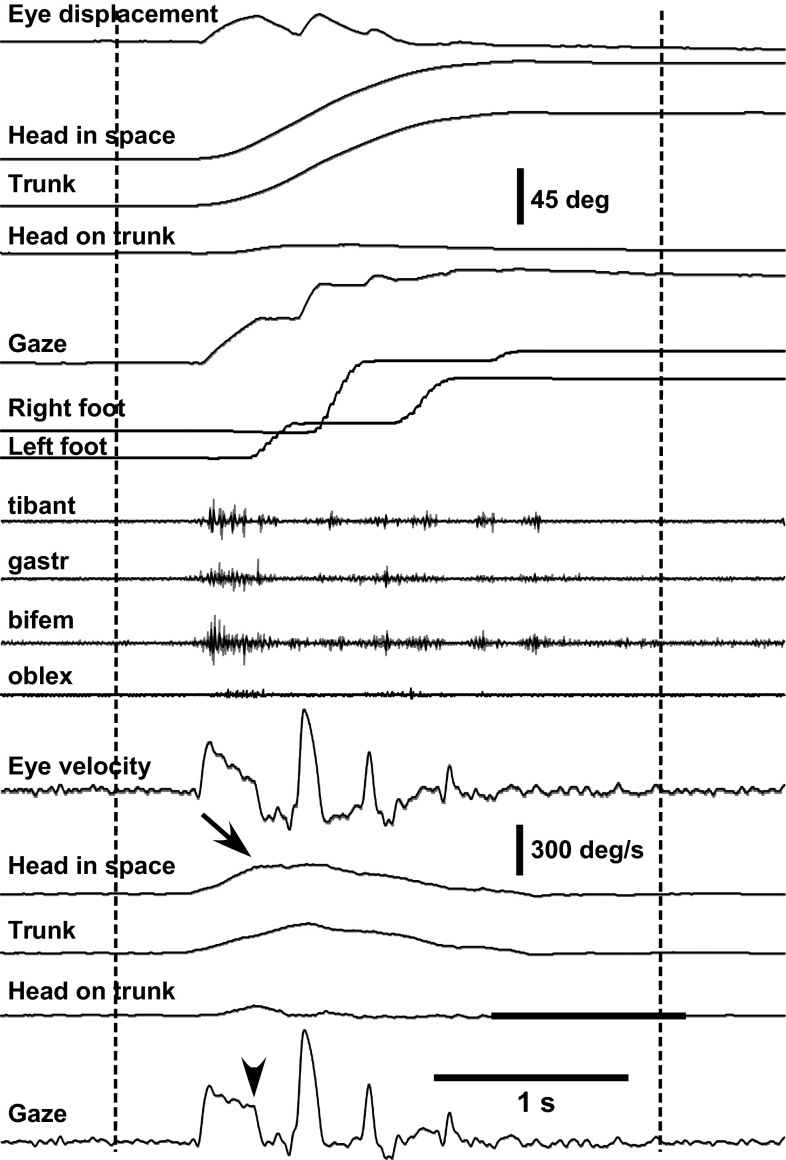
A rightward multiple-step gaze shift to the central remembered target at 135° offset in complete darkness. Target presentations and head/trunk positions adopted before and after the combined movement are as shown in the cartoon of Fig. 1. Extinguishment of the eccentric LED (‘go’ signal) and reappearance of the central LED are indicated by the *left* and *right vertical* markers, respectively. The premature termination of the initial gaze shift long before target acquisition (*arrowhead*, *bottom trace*) coincides with the emergence of a plateau of the up to this point increasing head-in-space velocity trace (*arrow*)

Head-in-space movement resulted from the sum of head-on-trunk and trunk rotation in space. The dynamic trajectory of head-in-space velocity was conspicuously monophasic, increasing smoothly to a peak and then decreasing slowly until realignment with the memorized gaze direction (deceleration phase usually somewhat longer, Figs. 1 and 2). Head-in-space peak velocity (Fig. 3a) and duration increased thereby as a function of target eccentricity (factorial ANOVA, *F* = 26.1 *p* < 0.0001 and *F* = 25.1 *p* < 0.0001, respectively). In contrast to head-in-space, the dynamic trajectories of trunk-in-space, and most particularly head-on-trunk motion, were more variable (Fig. 2). Just as peak head-in-space velocity and duration, the head-in-space acceleration phase was also significantly dependent on target eccentricity, i.e. it was shorter when moving to 90° targets and longer when reorienting to 180° targets (ANOVA, *F* = 18.0, *p* < 0.0001, Fig. 3b; compare left and right records Fig. 2). Head-in-space acceleration during this time interval was not different among the various target eccentricities (on average 1362 ± 742°/s^2^), confirming that the Ss generally tried to comply with the instruction ‘go as fast as you can’. Noteworthy, in reorientations made by means of the multiple-step pattern (Fig. 4), mean head-in-space acceleration was significantly low (on average 593 ± 310°/s^2^, i.e. less than half the acceleration observed when assembling single-step gaze shifts, ANOVA, *F* = 49.4, *p* < 0.0001). In many instances, the premature termination of primary gaze shift long before reaching the position of the centrally located remembered target was signalled by an abrupt deceleration break in the, up to this point, monotonically increasing head-in-space velocity trace (arrows in Fig. 4). As a result of these modifications, peak velocity was significantly lower during multiple-step than during single-step gaze shifts (172 ± 52 vs. 279 ± 80, 211 ± 64 vs. 363 ± 110 and 266 ± 54 vs. 428 ± 127°/s to targets of 90°, 135° and 180° eccentricity, respectively, *F* = 29.7, *p* < 0.0001). However, even if interrupted by variable periods of stationary gaze, the displacement of the visual axis was terminated again very close to the remembered target.

As already reported in the literature (reviewed by Leigh and Zee 2006) for smaller head-free gaze displacements, gaze saccade duration, though quite variable, correlated significantly and increased linearly with gaze amplitude in single-step gaze transfers (*n* = 181, *R* = 0.7, slope 3.2 ms/°). Single-step transfers were approximately 100–300 ms faster than multiple-step gaze displacements (315 ± 75, 449 ± 107, 599 ± 135 vs. 506 ± 78, 611 ± 132, 791 ± 138 ms to targets of 90°, 135° and 180° eccentricity, respectively, *F* = 82.8, *p* < 0.0001). Finally, head-in-space oscillations (quantified by the absolute ratio of the first negative peak to peak head velocity, on average 0.06 ± 0.03) did not increase with the amplitude of head-on-trunk movement (*R* ^2^ = 0.24).

**Fig. 5:**
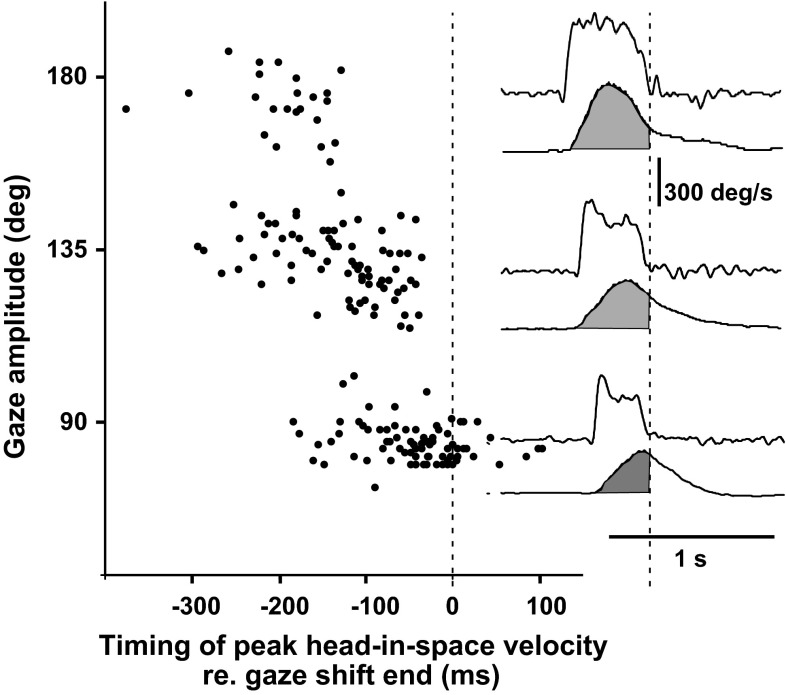
Timing of peak head-in-space velocity in relation to gaze shift end in fast single-step reorientations. Data points are plotted from *bottom* to *top* in order of increasing gaze shift amplitude. Head-in-space attains peak velocity well before gaze shift end (*vertical marker*). The time span between these events increases with gaze amplitude. This is exemplified for 90°, 135° and 180° reorientations (*lower*, *middle* and *upper* traces, respectively) on the *right*. The *shaded* area beneath the head-in-space velocity traces represents the contribution of head-in-space displacement to gaze amplitude

In order to examine whether head-in-space control is autonomous or driven by an ultimate goal-oriented function (in this case gaze reorientation), we examined how the timing of peak head-in-space velocity was associated with the end of the gaze shift in single-step movement patterns (Fig. 5). If head-in-space movement was solely controlled by gaze signals, it might be expected that, because of the forced instruction, head-in-space would continue to accelerate until arriving at the desired gaze position (final goal, vertical middle marker in Fig. 1) and then start to decelerate, as gaze shift end does not coincide with head-in-pace alignment with the remembered target. Note that in Fig. 5, however, peak head-in-space velocity does not coincide with gaze shift end; it occurs earlier and earlier as gaze shift duration and amplitude increase, suggesting that the control of head-in-space motion does not exclusively aim at conveying the line of sight as soon as possible to the target. In contrast, the duration of the ‘acceleration phase time’ increases with target eccentricity, implying that the latter is one of the factors dictating the timing of peak head-in-space velocity.

### Covariance of eye, head and trunk motion during single-step gaze transfers (principal component analysis, PCA)

**Fig. 6:**
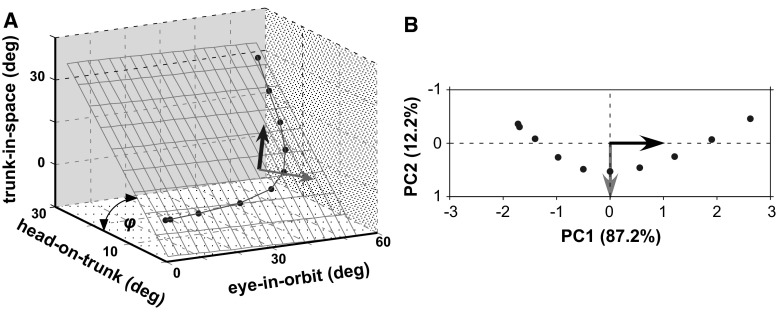
Covariation plane of eye-in-orbit, head-on-trunk and trunk-in-space displacements to 135° target eccentricity (time window 0–333 ms, each *black point* represents the mean value of 53 samples). Time progresses from the *left* to the *right*. The depicted plane in **a** has been calculated by multilinear regression. The *black* and *grey* orthogonal unity vectors stand for the direction of PC1 and PC2, respectively. The projection of the data samples on the plane can be visualized by the *x*/*y* scatterplot of PC1 versus PC2 mean scores in B

Mean ± standard deviations of percentages of variance accounted for by individual principal components (PCs) are shown in Table 1, separately for single-step gaze shifts to 90°, 135° and 180° targets. PCA was applied whenever sufficient numbers (*N* > 15) of trials for statistical analysis were available. The first PC was significant (Bartlett’s test, *p* < 0.001) and accounted for almost 90 % of variance in all analysis time windows. The second PC was also significant overall and accounted approximately for about 10 % of variance (*p* < 0.001). In contrast, the third PC was non-significant in all time windows and target eccentricities, accounting for <1 % of variance. Variance up to half a second after movement initiation could be thus accounted for by essentially the first two PCs (i.e. the sum of their percentages was approximately 100 %), suggesting that the three mechanical degrees of freedom under consideration are reduced to two kinematic degrees of freedom. Visualization of these relationships is exemplified in Fig. 6. Mean eye, head and trunk displacements from 53 trials to 135° targets up to 333 ms (i.e. 10 data samples) are plotted in the three-dimensional position space defined by the variables (eye-in-orbit; head-on-trunk and trunk-in-space). Time progresses from the origin of the coordinate system to the right, upward and backward (Fig. 6a). Two significant PCs imply that the data approximately vary in only two orthogonal directions defined by the first and second PCs, i.e. in a plane. The projection of data samples in this plane is illustrated in Fig. 6b (*x* and *y* axes give mean PC scores, units are normalized as a consequence of auto-scaling, c.f. “Materials and methods”). PC1 increases with time (from −1.7 to 2.6), while PC2 first increases (from −0.4 to 0.5) and then decreases to −0.5. All variables have positive mean PC1 weights (i.e. show the trend represented by PC1). Eye mean PC2 weight is again positive, while those of head and trunk are both negative. In consequence, head and trunk displacements covary in both directions described by the significant PCs.

An approximation of the orientation of the covariation plane, calculated from displacement data by means of multilinear regression, is depicted in Fig. 6a and described by the angles *θ* and *φ*. Note from Table 1 that *θ* is often close to 90° while *φ* is mostly larger than 45°. Because *θ is* mostly close to 90°, the covariation plane is approximately perpendicular (orthogonal) to the plane defined by the axes head-on-trunk and trunk-in-space. In consequence, head-on-trunk and trunk-in-space displacements are linearly correlated, i.e. they lay approximately upon a regression line and can be replaced by one variable, for example, head-in-space. The slope of this relationship is larger than unity as *φ* is mostly >than 45°. In this way, trunk-in-space is on average larger than head-on-trunk displacement throughout the gaze transfers. Of note, these relationships do not apply during ‘multistep’ (nystagmic) transfers. Here, three derived significant PCs account for the data variance (Sklavos et al. 2010).

### Onset of EMG activity in lower limb and trunk muscles

**Fig. 7:**
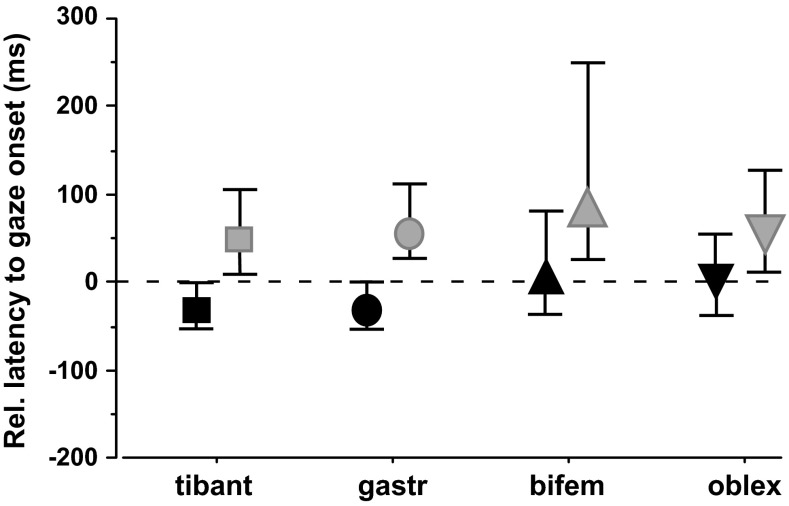
Latency of EMG activity relative to gaze shift onset. Median and interquartile ranges in the right tibialis anterior (tibant), gastrocnemius (gastr), biceps femoris (bifem) and obliquus externus (oblex) muscles. Gaze onset is indicated by the *horizontal dotted line*. Only those rightward rotations are included during which stepping started with the right foot. While median EMG onset precedes or coincides with eye rotation when the location of the target is known (inbound or return trials, *black quadrangle*, *circle* and *triangles*), it follows the rotation onset in outbound, unpredictable trials (same notation in *grey*)

Ss activated the right anterior tibial and gastrocnemius muscles approximately 30 ms on average before starting to rotate gaze (i.e. the eye) rightwards to the centrally located remembered target (Fig. 7, black quadrangles and circles; also Fig. 1). Biceps femoris and lateral oblique (black triangles) were activated approximately 40–50 ms later than eye movement onset. Note the larger scatter of values of onset of EMG activity in these proximal muscles. When the location of the target was not known beforehand, i.e. in the unpredictable outbound trials, both distal and proximal muscles were activated mostly after the initiation of eye rotation (grey quadrangles, circles and triangles, *p* < 0.003 Mann–Whitney *U* test, inbound vs. outbound). There was not any statistical difference in the onset of EMG activation from the right tibial and gastrocnemius muscles, both for inbound and for outbound reorientations, i.e. they were activated on average synchronously. Not unexpectedly, EMG activity in these muscles decreased as the right foot started to rotate rightwards.

## Discussion

More than 70 % of reorientations to the centrally located remembered target in this study were accomplished by rapid, accurate single-step movement patterns (in contrast to our previous studies at free speed where single-step gaze shifts were observed in approximately 30 % of cases; Anastasopoulos et al. 2009). *S*s were instructed to move as fast as possible and were thus able to attain high head and trunk velocities. In the remaining 30 % of trials, however, the progression of the line of sight was interrupted by one or more variable intervals of stable gaze (multiple-step, nystagmic or ‘scanning’ pattern). All the same, gaze displacement ended again close to the remembered target. Multiple-step gaze shifts were characterized by significantly low head-in-space velocity. High head-in-space velocity during task execution thus increases the probability of eliciting single-step gaze shifts and quickly acquiring a predetermined direction of the visual axis, as hypothesized in previous studies (Anastasopoulos et al. 2009, 2011, 2013). The slower, multiple-step gaze shift pattern, incorporating sections of VOR mediated gaze stability, looks suitable for visually scanning the environment during lower velocity roaming head movements.

Subjects confronted with the task to acquire the target as soon as possible quickly increased the velocity of the head-in-space to a peak before decelerating up to the target. Statistical evaluation of segmental spatial covariation by PCA confirmed that eye, head and trunk displacements are coupled at the kinematic level such that their motion can be controlled until gaze shift end with a two degrees of freedom system (i.e. eye- and head-in-space). This presumably presents the CNS with a simpler control task.

### Separate control of head-in-space movement

A conspicuous, central feature of large, single-step reorientations to remembered targets was the high peaked monophasic head-in-space velocity traces (Fig. 2), suggesting that head-in-space rotation is related to control signals switched from maximum ‘on’ to maximum ‘off’ (‘bang–bang’ double pulse). This was a consequence of the forced instruction to the Ss and indicates that inertial characteristics of the head/trunk mechanical system predominate. Head-on-trunk and trunk-in-space movements were, instead, more variable both during the accelerating and decelerating phase (Fig. 2; also before and after gaze shift end). Further evidence suggestive of a separate or independent head motion controller is the fact that time to peak velocity and the magnitude of peak velocity for the ‘composite’ head-in-space variable increased with target eccentricity (Fig. 3). Similar ‘main sequence’ relationships have been earlier described for trunk restrained primates (Tomlinson and Bahra 1986) or cats (Guitton et al. 1990), i.e. when head-on-trunk equals head-in-space movement. Zangemeister et al. (1982) studied time-optimal head movements in sitting humans and found that the duration of head motion was independent of movement amplitude. In contrast to the results of Zangemeister et al., head movement in our study appears to be controlled by the duration of force generation rather than by the amount of force (Fig. 3), probably because of the large target eccentricities and the large trunk inertia involved. The head control system is, in the current experiments, clearly functioning close to the upper limit of force generation, and this strict demand may be responsible for the fact that Ss fail to acquire in all occasions distant targets with single-step gaze shifts (see below). Under the requirement of maximal effort in executing a heavy task, it is conceivable that Ss had to prolong the application of force and consequently failed more frequently with increasing target eccentricity.

A further salient finding of our study in favour of separate control for head-in-space is that command timing does not relate to gaze displacement but, instead, head-in-space displacement (Fig. 5). If the head was exclusively under gaze control, a continual increase in head-in-space velocity up until gaze shift end would be expected. Our findings contribute to the ongoing debate whether it is gaze or its individual eye and head components that are controlled during orienting behaviours (Guitton et al. 2003; Choi and Guitton 2006; Freedman 2001; Sylvestre and Cullen 2006) with evidence supporting the view that the controller for gaze and head-in-space movement may not be identical. The neural mechanism underlying the control of the large number of muscles and joints of the neck and trunk, such that a solution appropriate for the task in hand (i.e. time-optimal displacement of head-in-space) can be achieved, remains to be elucidated. Notably, single-step displacements are characterized on average by linear correlation of head-on-trunk and trunk-in-space displacements throughout the gaze transfer. Moreover, trunk displacement is on average larger than head-on-trunk displacement throughout visual axis transfer. Multiple-step movements do not fulfil these kinematic constraints as they do not demonstrate reductions in significant PCs and covariation among the segments (Sklavos et al. 2010). The present results may facilitate future investigations on the control mechanism. They have to be interpreted, however, with caution as Ss were required to reestablish the same body position, and this may have encouraged coordinated, en-bloc movements, reducing variability. The study of more complex or ecological tasks in the future may refine our results and reveal further parameters of segment covariation.

Why did Ss frequently choose to move head-on-trunk far below 60–70° (i.e. the limit of the so-called neutral zone, McClure et al. 1998) though they thus increase the duration of head-in-space movement up to the target (Fig. 2)? Our results show that this strategy does not reduce unwanted head-on-trunk oscillations (Saglam et al. 2011). It possibly represents an anticipatory mechanism aiming at reducing the amount of control energy required to rotate head-on-trunk to more eccentric positions due to concomitant increase in the applied force (Kardamakis and Moschovakis 2009). Contrary to these feed-forward models, we suggest, a substantial contribution from feedback control, based mainly on vestibular input, because the movement of the support (i.e. the feet) is extremely variable and subject to many possible disturbances (Sklavos et al. 2010). It may also be easier to coordinate the many segments and achieve a well scaled and timed head in space velocity, as our Ss did managed to do, if afferent online velocity information (=vestibular input) is available. This question could be answered by testing patients with bilateral vestibular failure during whole-body gaze transfer task, and preliminary data in two such patients support this view (ongoing project).

### The control of eye movement and constraints on gaze shift duration

Separate control signals for the eye and head have been proposed by several researchers because of the variability in the relative timing of the start of eye and head movements and the effect of initial eye-in-orbit position on the contribution of the eyes and head to gaze displacement (for a review, see Freedman 2008). Separate control for the most distal and proximal parts of linked systems has been modelled recently by Daye et al. (2014). According to this scheme, the most distal subpart (here the eye) of such a ‘hierarchical’ structure is governed only by global goal feedback (here gaze). Indeed, the relationships between amplitude, velocity and duration of the eye-in-orbit movement of large single-step gaze displacements were not stereotyped but depended on how the head moved. For example, reaccelerations of the eye-in-orbit motion after the peak of head-in-space velocity may have been generated to correct deviations of the progressing gaze trajectory from the efference copy of ongoing motor commands and complement the separately progressing head motion before the impending gaze shift termination (Fig. 1). Remarkable accuracy in darkness (>90 %) was achieved in spite of the variable head-in-space contribution even for multiple-step displacements. We suggest that a mechanism monitors continually overall visual axis progression and modifies accordingly the eye component, given that head-in-space movement has to obey bang–bang control appropriately for a certain target eccentricity.

The fact that high head-in-space velocity and single-step gaze shifts are interconnected makes sense biologically when target acquisition occurs under ‘as soon as you can’ conditions, but how are these two variables associated mechanistically? It is clear from our findings that reemergence of the VOR and gaze stabilization does not depend solely on gaze error, as most gaze models propose (cf. Galiana and Guitton 1992), but they are related to the strength of the descending signal moving head-in-space, i.e. the command sent to agonists moving ‘head-in-space’. In multiple-step gaze shifts, characterized by premature termination of ‘saccadic mode’ in spite of the instruction, average head-in-space acceleration was less than half of that during single-step reorientations (cf. “Results”, compare Fig. 1 with Fig. 4). We argue that high head-in-space acceleration results both in adequately fast head-in-space movement, contributing quickly to approximately 45°, 90° and 135° of displacement for targets at 90°, 135° and 180°, respectively (grey area under the velocity traces, given that eye rotation covers utmost 45–50° on average, Fig. 5) and in prolongation of the time interval available for ‘saccadic mode’. The neural mechanism that determines the duration of ‘saccadic mode’ and the termination of gaze or eye saccades is largely unknown. It has been hypothesized that descending signals from the rostral superior colliculus and/or frontal eye fields (Bergeron and Guitton 2002; Hanes et al. 1998; Segraves 1992) gate omnipause neuron activity (which resumes at saccade end) and by this disinhibit gaze bursters in the pons allowing for gaze displacement. Electrical stimulation of the omnipause neurons brainstem region in primates stops ongoing gaze shifts in mid-flight (Gandhi and Sparks 2007). During the stimulation, interval gaze position remains constant (‘stabilization mode’) and, while head movement continues along its trajectory, the eye counter-rotates with a roughly equal velocity in the opposite direction (i.e. the VOR is reactivated). Still, mechanisms other than OPN discharge may contribute to saccadic termination. The oculomotor cerebellum has been also implicated in the termination of rapid gaze displacements (Fuchs et al. 2010; Rucker et al. 2011). We suggest a straightforward, mechanistic explanation of our findings, i.e. that the gating period of this putative mechanism is too short if the descending signals accelerating head-in-space are weak. The stronger demands imposed on the duration of head-in-space movement due to the large eccentricities and large segmental inertia revealed the conditional ability of the gaze control system to delay beyond a top limit switching from ‘saccadic mode’ to ‘stabilization mode’. The mechanism is thus not uniquely controlled by a gaze-motor-error signal (cf. Paré and Guitton 1998). Still, the experimentally observed relationship between the strength of the ‘head-in-space’ neural control signal and the duration of the ‘saccadic mode’ interval may be not a causal one; the reverse may be also true, i.e. head-in-space velocity is simply low when multi-step displacements are planned, e.g. when a visual scanning pattern is called for.

### Stabilization of the ankle joint precedes eye rotation to the target

Foot rotations in this experimental paradigm are extremely variable and start mostly considerably later than movements of the eye, head and trunk (on average 350 ms later than the eye, Anastasopoulos et al. 2009). As foot latency is significantly reduced when target location is known (i.e. in return trials), it has been previously hypothesized that proprioceptive input and local mechanisms (i.e. from hip stretch) are overruled by supraspinal inputs and that stepping initiation is thereby controlled more rostrally. On the other hand, segmental movement variability analysis has previously indicated that that lower extremities contribute initially to postural rather than gaze displacement control (Sklavos et al. 2008). Indeed, our current results suggest that the turning synergy to predictable targets is initiated with the synchronous activation of antagonistic leg muscles. Stiffness of the ankle joint thus increases, and we hypothesize that forces involved in trunk rotation will less likely threaten balance. More detailed measurements are, however, required in this direction. Also, EMG activity in muscles that rotate the foot may be expected to ensue, but this was not recorded and cannot be answered with certainty. Such ankle stabilization is likely part of an anticipatory postural adjustment in preparation of a complex movement sequence (Assaiante et al. 2000), in this case ultimately aiming to shift the eyes in space. Given that in conventional head-fixed saccadic experiments, anticipatory adjustments and assemblage of multisegmental synergies are not required, our EMG findings thus agree with the view that the long eye latencies observed in this paradigm relate to active delaying of the eye saccades until all anticipatory postural adjustments and segments are ready to go (Scotto Di Cesare et al. 2013).
